# Predictors of impending acute chest syndrome in patients with sickle cell anaemia

**DOI:** 10.1038/s41598-020-59258-y

**Published:** 2020-02-12

**Authors:** Salam Alkindi, Ikhlas Al-Busaidi, Bushra Al-Salami, Samir Raniga, Anil Pathare, Samir K. Ballas

**Affiliations:** 1 Department of Haematology, Sultan Qaboos University Hospital, Muscat, Oman; 2 College of Medicine & Health Sciences, Muscat, Oman; 3 Department of Radiology & Molecular Imaging, Sultan Qaboos University Hospital, Muscat, Oman; 4 Cardeza Foundation for Hematologic Research, Thomas Jefferson University, Philadelphia, USA

**Keywords:** Outcomes research, Risk factors

## Abstract

Acute chest syndrome (ACS) is a major complication of sickle cell anaemia (SCA) and a leading cause for hospital admissions and death. We aimed to study the spectrum of clinical and laboratory features of ACS and to assess the predisposing factors and predictors of severity. A retrospective case-control cohort was studied by retrieving patient information from electronic medical records after ethical approval. One hundred adolescents and adults with SCA and hospital admissions for ACS were identified through the discharge summaries, along with 20 additional patients presenting with VOC, but without ACS (controls). Among the patients with ACS, fever (>38.5 °C), reduced oxygen saturation (<95) and asplenia significantly differed when compared to those of controls (p < 0.05, chi-squared test). The degree of severity was reflected in the use of non-invasive ventilation (NIV), simple and exchange transfusions, and the presence of bilateral pleural effusions and multi-lobar atelectasis/consolidation, which were significantly higher in the cases with ACS than in the controls. Lower haemoglobin (Hb) and high WBC counts were also significantly different between the two groups (p < 0.05, Student’s t test). Using logistic regression, our study further demonstrated that asplenia, fever, and reduced O_2_ saturation, along with low Hb and leukocytosis, were important predictors for the development of ACS.

## Introduction

Sickle cell anaemia (SCA) is an autosomal recessive disorder characterized by a point mutation in codon 6 of the beta globin chain, where glutamic acid is replaced by valine, resulting in the formation of HbS with varied clinical manifestations^1^. The abnormal Hb is insoluble and polymerizes when exposed to low O_2_ tension, leading to the major clinical manifestations of SCA, including recurrent vaso-occlusive crisis (VOC), predisposition to infections and reduced red cell survival with anaemia^2^.

Sickle cell disease (SCD) is prevalent in sub-Saharan Africa and the Indian sub-continent, but human population mobility has spread the disorder throughout Europe, Asia and the Americas^3,4^. SCD is highly prevalent in Oman, with an estimated sickle cell gene frequency of 5.8% and a prevalence of sickle cell traits and SCA in Omani children of 4.8% and 0.3%, respectively^5,6^. It is thus estimated that one neonate of every 323 newborns in Oman has SCA^6^.

Acute chest syndrome (ACS) is defined as the presence of fever and/or new respiratory symptoms accompanied by the presence of a new pulmonary infiltrate on chest X-ray and is one of the most important causes of death and the second most common cause of hospitalization in SCA patients^7^. In a retrospective study, ACS was found to be responsible for 69.6% of SCA ICU admissions and was associated with a 16% mortality rate^7^. Furthermore, ACS is associated with a high risk of sickle cell-related mortality and morbidity, including prolonged hospitalization^8^. Although the aetiology of ACS is multifactorial, the Cooperative Study of Sickle Cell Disease (CSSCD) in the United States identified several aetiological factors, including infections (bacterial, viral and others), thrombosis, fat and pulmonary embolism, but in 50% of the cases, a clear aetiology could not be identified^9^. Furthermore, in the same study, high Hb, low HbF and high WBC counts during steady state were identified as risk factors for the development of ACS. It is also well known that many patients with SCA develop ACS after being admitted to the hospital^9^. Styles *et al*.^10^ observed that secretory phospholipase A2 (sPLA2) demonstrated a 67% specificity with 100% sensitivity to predict impending ACS in the patients they studied. This observation was further substantiated by Ballas *et al*.^11^ in 80% of SCA patients in their study. However, Styles *et al*., in a more robust randomized study design, demonstrated a 71% specificity with 73% sensitivity, although the positive predictive value of sPLA2 was only 24%^12^. Unfortunately, the test is not yet available commercially. Therefore, the aim of this study was to evaluate the clinical and laboratory features of patients with ACS compared to patients with simple VOC and to assess the risk factors predisposing the development of ACS and potential predictors of its severity.

## Materials and Methods

This is a single institution case-control cohort study of SCA patients, who were admitted with ACS between July 2009 and June 2017 at our institution. A protocol was submitted to the Medical Research and Ethics Committee (MREC) to collect relevant details of consecutive cases from the medical records of patients who were admitted for ACS. All patients were Omani Arabs, above 15 years of age, with SCA (HbSS and HbSβ°), whereas other SCD subtypes, such as SD, SC, and SE, were excluded. Ninety-two SCA patients (100 episodes) were identified through the discharge summaries as having had ACS, and 20 SCA patients with simple VOC (20 episodes) who did not have ACS were used as the controls during the same time frame. The controls had an acute episode of severe pain in spite of the administration of analgesic medication and needed admission without any respiratory symptoms. These controls were selected consecutively in a 1:5 proportion during the same time frame as the enrolment of cases. Acute chest syndrome was defined as patients presenting with fever and/or respiratory symptoms, accompanied by new infiltrates on the chest radiograph. All methods were carried out in accordance with relevant local guidelines and regulations. The MREC approval (MREC #1333) was for a retrospective data collection and analysis; hence, individual patient consent was not required/mandated by the MREC.

The study evaluated clinical and laboratory parameters, including the vital signs such as heart rate, blood pressure, respiratory rate and pulse oximetry O_2_ saturation, for both groups of patients throughout the whole period of admission. Other clinical parameters that were studied included fever, chest/back pain, cough, wheezing, crepitation and use of non-invasive ventilation (NIV). The laboratory parameters included Hb, HbF, reticulocyte count, WBC count, platelet count, serum LDH, AST, ALT, serum bilirubin, serum creatinine, and CRP. Blood culture studies and the use of hydroxyurea (HU) and simple and exchange transfusion were also recorded. Radiological investigations recorded findings using chest X-rays, abdominal ultrasounds and chest CT scans.

### Statistical analysis

The Statistical Package for Social Science (IBM SPSS, USA ver.23) was used to analyse the collected data. Normally distributed data were characterized as means with standard deviations, whereas data that were skewed [i.e., not normally distributed] were characterized as medians with interquartile ranges (IQRs) for continuous variables and as percentages and frequencies for categorical variables. The chi-squared test was used to study the statistical significance of qualitative variables, whereas Student’s t test was used to study the differences in the quantitative parameters between the cases and controls. Logistic regression with odds ratios and 95% confidence intervals (CIs) was performed to ascertain the effectiveness of the predictors of ACS. A p-value < 0.05 was considered statistically significant.

## Results

Although there was a similar frequency of males in the cases and controls, males tended to suffer from ACS at an earlier median age than females (27 years compared to 31 years in females), but this was not statistically significant. History of previous ACS and hydroxyurea therapy was also similar between the two groups (Table 1).

**Table 1:** Comparison of demographic, clinical and laboratory parameters for SCA patients with ACS and without ACS.

Parameters	Cases (92)	Controls (20)	P value
**Demography**			
Median age in years (IQR)	28(7.75)	26(6.25)	0.432^#^
Sex, Total cases, n, M: F	63:29	12:8	
Median age in years, males (IQR)	27(7)	—	
Median age in years, females (IQR)	31(5)	—	
**Clinical Features:**			
Prior ACS, n(%)	54(58.7)	11(55)	0.761^#^
HU therapy, n(%)	51(55.4)	10(50)	0.634^#^
Chest/Back pain, n(%)	83(90.2)	15(75)	0.062^#^
Fever [>37.80°C], n(%)	81(88)	13(65)	0.01^#^
Cough, n(%)	38(41.3)	6(30)	0.319^#^
Tachycardia [>100], n(%)	74(80.4)	14(70)	0.296^#^
Hypotension [<80], n(%)	71(77.2)	14(70)	0.542^#^
Tachypnea [>25], %	25(27.2)	1(5)	0.03^#^
**Reduced Pulse oximetry**			
O2 saturation [<95], n(%)	67(72.8)	5(25)	0.0001^#^
Crepitations, n(%)	52(56.5)	0	0.000^#^
Wheeze, n(%)	10(10.9)	0	0.121^#^
**Radiological Findings:**			
Asplenia, n(%)	51(55.4)	3(15)	0.001^#^
Pleural effusion n(%)	76(82.6)	0	0.000^#^
On X-ray chest, Mild n(%)	64(84.2)		
Moderate n(%)	12(15.8)		
On CT Scan, n(%)	49(53.3)		
Multilobar atelectasis on CT scan, n(%)	83(90.2)	0	0.000^#^
Multilobar atelectasis on X Rays, n(%)	56(60.8)	0	
**Laboratory Parameters:**			
Median Hb nadir, g/L [95% CI]	7.8[7.6–8.1]	9.1[8.3–9.7] 0.0007^$^	0.0007^$^
Median WBC count basal, X10^9^/L [95% CI]	9.4[9.3–10.9]	7.4[7.1–9.7] 0.058^$^	0.058^$^
Median WBC count Max, X10^9^/L [95% CI]	17.6[16.8–19.3]	10.2[10.1–14.1] 0.0001^$^	0.0001^$^
Median platelet count basal, X10^9^/L [95% CI]	382[354–416]	343[226–459]	0.356^$^
Median platelet count nadir, X10^9^/L [95% CI]	227[219–283]	175[180–391]	0.439^$^
Median retic count, % [95% CI]	5.5[5.3–8.0]	4.7[4.2–4.9]	0.206^$^
Median basal HbF, % [95% CI]	7.0[6.9–90.]	7.6[6.5–11.7]	0.441^$^
S.LDH at admission, u/L [RR 135–225]	745[542–872]	487[356–619]	0.131^$^
S.LDH max, u/L	1030[658–1287]	569[410–708]	0.09^$^
CRP at admission, mg/L [RR 0–5]	43[42–95.5]	71.5[49.7–104.7] 0.962^$^	0.962^$^
CRP max, mg/L	180[153–203]	107[78.9–159.7] 0.066^$^	0.066^$^
AST, u/L [RR 0–40]	49.7[49.3–66.4]	38.8[33.3–46.1] 0.127^$^	0.127^$^
ALT, u/L [RR 0–41]	36.6[36.1–62.4]	30[25.2–46.9] 0.373^$^	0.373^$^
S. bilirubin, μg/ml [RR 0–17]	44.2[44.1–66.6]	35[28.1–45.2]	0.056^$^
**Interventions:**			
**Use of NIV, n(%)**	15(16.3)	0	0.047^#^
Abnormal blood cultures, n(%)	4(4.3)	0	0.872^#^
Simple blood transfusions, n(%)	58(63)	4(20)	0.016^#^
Exchange blood transfusions, n(%)	61(66.3)	2(10)	0.001^#^

Key: ^#^-chi-squared test; ^**$**^-unpaired Student’s t test; IQR- interquartile range, RR- reference range, HU- hydroxyurea, ACS- acute chest syndrome, Hb- Haemoglobin, WBC- White blood cell count, NIV - non-invasive ventilation, LDH-lactic dehydrogenase, CRP- C-reactive protein, AST- aspartate transaminase, ALT- alanine transaminase.

Fever, crepitation and reduced O_2_ saturation by pulse oximetry were significantly abnormal in the cases with ACS (p < 0.05, chi-squared test). This was also reflected in the significantly higher odds ratios in the cases compared to the controls (p < 0.05). Radiologically, abdominal ultrasound demonstrated a significantly higher degree of asplenia in the cases than in the controls (p < 0.05, chi-squared test). Pleural effusions, positive chest X-rays and CT scans of the chest as well as multilobar atelectasis were significantly greater in the cases than in the control (p < 0.05, chi- squared test).

Complete blood counts showed that Hb nadirs and maximal WBC counts showed a statistically significant difference between the two groups (p < 0.05, Student’s t test) with logistic regression analysis also confirming the significance (Table 2). HbF levels did not show any significant differences between the cases and controls. Furthermore, among the biochemical parameters, although serum CRP, serum total bilirubin, and serum LDH levels were elevated, they did not reach statistical significance (Table 1).

**Table 2:** Logistic regression analysis with odds ratios for the demographical, clinical & laboratory parameters of SCA patients with ACS and without ACS.

Parameters	Odds Ratio	95% CI		P value
**Demography**				
Age	1.087	0.978	1.208	0.123
Sex (Male)	1.135	0.425	3.032	0.8
**Clinical Features:**				
Prior ACS	1.067	0.615	1.851	0.753
HU therapy	1.109	0.605	2.032	0.658
Chest/Back pain,	3.074	0.899	10.68	0.074
Fever [>37.80°C],	3.965	1.316	11.85	0.014
Cough,	1.690	0.609	4.742	0.321
Tachycardia [>100],	1.366	0.678	2.774	0.389
Hypotension [<80],	1.013	0.988	1.039	0.098
Tachypnea [>25],	4.226	0.896	19.74	0.068
**Reduced pulse oximetry**				
O2 saturation [<95],	8.111	2.689	24.47	0.000
Asplenia,	7.049	1.957	18.68	0.000
**Laboratory Parameters:**				
Median Hb basal,	0.803	0.555	1.161	0.244
Median Hb nadir,	0.535	0.385	0.805	0.003
Median WBC count basal,	1.175	0.991	1.393	0.064
Median WBC count max,	1.236	1.098	1.392	0.000
Median platelet count basal,	1.002	0.998	1.005	0.353
Median platelet count nadir,	0.999	0.996	1.002	0.438
Median retic count,	1.190	0.897	1.579	0.229
Median basal HbF,	0.983	0.875	1.060	0.439
S.LDH at admission,	1.002	1.000	1.004	0.132
S.LDH max,	1.001	1.000	1.003	0.107
CRP at admission	1.000	0.994	1.007	0.964
CRP max	1.005	1.000	1.010	0.071
AST, u/L	1.024	0.995	1.054	0.103
ALT, u/L	1.010	0.990	1.031	0.331
S. bilirubin,	1.024	0.998	1.051	0.069
**Interventions:**				
Simple blood transfusions,	8.514	2.310	31.375	0.001
Exchange blood transfusions,	31.571	4.031	247.26	0.001

Key: HU- hydroxyurea, ACS- acute chest syndrome, Hb- Haemoglobin, WBC- white blood cell count, NIV- non-invasive ventilation, LDH-lactic dehydrogenase, CRP- C-reactive protein, AST- aspartate transaminase, ALT- alanine transaminase.

The blood cultures were positive in 4% of the cases compared to none in the controls. Furthermore, the use of NIV was reported in 16.3% of the cases compared to none in the controls.

Sixty-three percent of patients in the case group received simple blood transfusions compared to 20% in the control group, whereas exchange transfusions were given in 66.3% of ACS patients compared to 10% of the controls (p < 0.05, chi-squared test). The transfusions were based on the clinical evaluations and therapeutic decisions of the treating physicians. Only one death was recorded in the ACS patients compared to none in the control group.

## Discussion

SCA is the most prevalent hereditary haemoglobin disorder in Oman^5,6,12,13^. Furthermore, ACS is the leading cause of death among patients with SCA^7–9^. It is characterized by acute onset of chest pain, associated with tachypnea, fever, reduced oxygen saturation, dyspnea, cough, elevated WBC counts and not infrequently, with a decreased Hb level and new infiltrates on chest X-ray. Many patients with SCA are admitted with VOCs and progress to develop symptoms and signs of ACS^12^, as was observed in this study.

Our aim was to study the risk factors that lead to the development of ACS, as determining the risk factors will help in identifying patients at risk early, thereby directing special attention towards them to prevent adverse outcomes. We observed that patients were more likely to develop ACS if they were older and female compared to the control cohort admitted with a typical VOC. ACS is more common in children, but our study only included adolescents and adult patients. It is interesting that the median age of the study group was older than that of the control group, although not statistically significant. A previous history of ACS is a known potential risk factor, and it was seen in 58.7% of the ACS group; the mortality in this group was notably low (1.1%).

It is also known that males are at an increased risk for VOC compared to females^14–16^. Our study showed findings similar to these previously reported observations that ACS was more common among men than women. However, the reason for this observation is not clear. Gladwin MT *et al*.^16^ reported slightly higher HbF levels in females compared to male SCA patients and concluded that this could be protective, forming the basis for gender differences in nitric oxide bioavailability. Ikuta T *et al*.^17^ have shown that nitric oxide is linked to transcriptional control of HbF and may explain the link between the two, i.e., the increased prevalence of ACS in males. They believe that oestrogen facilitates nitric oxide production and limits its consumption. We were not able to test this hypothesis as the use of hydroxyurea therapy was seen equally in both cases and controls and would be a confounding bias affecting the association between drug exposure and outcome.

Asthma is known to increase ACS, especially in children, and increase the risk of recurrence^18,19^; however, none of our ACS patients gave any previous history of childhood asthma. Furthermore, although wheezing was higher in the ACS group than in the control group, it did not reach statistical significance, and a larger sample may be necessary to see any correlation^19^.

Vichinsky EP *et al*. reported that many patients develop ACS following admissions with VOCs^9^. In this study, although many patients presented with features of ACS, some patients presented initially with back/chest pain, and these individuals were found to be more prevalent amongst those who progressed to ACS than among the controls in this study. However, this result did not reach statistical significance. Nevertheless, fever, hypoxia, and abnormal lung findings on clinical examination at admission are significant predictors for evolving to full blown ACS^9^. These findings also reemphasize the observations made in other studies, including the corporative study on SCA and others^20,21^.

As reported previously^20,21^, a higher WBC count at baseline predicted a higher risk of ACS and was also seen in our study. Furthermore, these patients demonstrated a further rise in WBC count associated with a considerable drop in haemoglobin from baseline and with significantly higher platelet counts, as these patients more frequently progressed to ACS when compared to VOC patients in the control group. The reasons for the association between a high leukocyte count and ACS incidence are not clear, but may relate to the underlying inflammatory process. Castro O *et al*.^20^ reported that higher leukocyte counts correlated with higher mortality rates. Therefore, the association of leukocytosis with ACS incidence could reflect the fact that both high WBC counts and high ACS rates are more frequently seen in patients with severe SCD^20^. The rise in serum CRP and the drop in Hb levels may signify increased haemolysis, sequestration in the lung or possibly sepsis. Furthermore, although the baseline HbF was relatively high in both groups, it did not seem to exert any protective effect on this group of patients, contrary to results from previous studies^20^.

Our study demonstrated significantly high serum LDH in ACS patients on the day of admission. This was also observed in the CSSCD and the French study on ACS in adults with SCA^20,22^. Furthermore, there was a trend towards significance with rising serum LDH max and serum bilirubin, which had p values of 0.09 and 0.05, respectively. This is consistent with the degree of haemolysis and underlying tissue necrosis, including bone marrow infarction, observed in these cases. Other biochemical parameters, such as serum AST and ALT, did not show any statistical significance in patients who had ACS.

There was a significantly higher prevalence of asplenia in the ACS group (55.4%) compared to the control group (15%). In view of the fact that HbF was similar in both groups, this is a significant observation. Furthermore, these asplenic patients are at an increased risk of sepsis and thrombosis, which may explain the elevated WBC counts and serum CRP in the ACS group. Although only 4% of the ACS group showed culture-proven bacterial infection, all of our patients received empiric broad-spectrum antibiotics. Infection is a major cause of morbidity and mortality in patients with SCA^9,19,21^. Our data are also consistent with a previous prospective study suggesting that bacterial pneumonia is not frequent in adult episodes of ACS^22^. Furthermore, in the CSSCD study from the USA, only a few bacteria were detected in lung samples on bronchoscopy, considering that bronchoscopy was invariably performed after empiric antibiotic administration, as is the universal practice^20^.

Radiological evaluation showed that the initial chest X-rays were normal in 21% of cases (data not shown), although they were abnormal in all the cases as ACS evolved in this group. Pleural effusions were seen in 76 (82.6%) of the cases on their chest X-ray and in 49 (53.3%) on their CT scan. Among these 76 cases, 12 (15.8%) had moderately severe effusions, while 64 (84.2%) had mild effusions. Multilobar atelectasis was observed in 60.8% and 90.2% of the cases on plain chest X-rays and CT scans, respectively. Not all patients had CT scans, as it is generally only requested in the most severe cases, which may explain the discrepancies in the findings between the two modalities.

Transfusions are often recommended for the treatment of ACS, and in a large American study^9^, transfusions were given in 75% of episodes; however, no controlled studies have been performed to evaluate the effect of transfusion on lung function and mortality. Additionally, transfusion is not devoid of serious side effects, including pulmonary oedema, blood-borne infections, and alloimmunization^23^. Nonetheless, simple and exchange transfusions are considered to be lifesaving in ACS patients as they improve oxygenation and reduce sickled RBCs and have other possible potential advantages. In our study, the majority of patients with ACS received simple (63% vs 20% in the control) and/or exchange transfusions (66.3% vs 10% in control).

A total of 16.3% of our patients needed NIV/mechanical ventilation, which is indicative of increased disease severity in these patients. Importantly, another study from our institution showed that 69.6% of all SCA patients admitted to intensive care had ACS^7^. It was also reported that the need for inotropic support and mechanical ventilation was a predictor of mortality in these patients^7^. Previously, mechanical ventilation was found to predict mortality and the increased utilization of hospital resources^18^.

This study has some shortcomings, including being retrospective, being from a single institution, and having a relatively small sample size. Large prospective studies are needed to validate the role of these risk factors to estimate their predictive value. Validating and confirming these findings may form the basis for creating a score for patients with VOC who are likely to develop ACS during their initial assessment at the time of admission.

## Conclusion

Our study demonstrated that high fever, reduced O_2_ saturation, and asplenia were associated with a high probability for predicting ACS during VOC episodes. Additionally, a significant drop in Hb and leukocytosis were also important predictors for the development of ACS in this study. Pleural effusion on chest X-ray, multi-lobar consolidation on CT scans; NIV and simple/exchange transfusions indicated severity of ACS in the Omani patients in this retrospective chart review study.

## References

[1] Bunn HF (1997). Pathogenesis and treatment of sickle cell disease. N. Engl. J. Med..

[2] Ballas SK (2010). Investigators, Comprehensive Sickle Cell Centers. Definitions of the phenotypic manifestations of sickle cell disease. Am. J. Hematol..

[3] Steinberg MH (2009). Genetic etiologies for phenotypic diversity in sickle cell anemia. Sci. World Journal..

[4] Knight-Madden JM, Hambleton IR (2016). Inhaled bronchodilators for acute chest syndrome in people with sickle cell disease. Cochrane database systemic reviews..

[5] Al Riyami AA, Suleiman AJ, Afifi M, Al-Lamki ZM, Daar S (2001). A community-based study of common hereditary blood disorders in Oman. East. Mediterr. Health J..

[6] Alkindi S (2010). Forecasting Hemoglobinopathy Burden Through Neonatal Screening in Omani Neonates. Hemoglobin..

[7] Tawfic Q (2012). Adult Sickle Cell Disease: A Five-year Experience of Intensive Care Management in a University Hospital in Oman. SQU Med. Journal..

[8] Jain S, Bakshi N, Krishnamurti L (2017). Acute Chest Syndrome in Children with Sickle Cell Disease. Pediatr. Allergy Immunol. Pulmonol..

[9] Vichinsky E (2000). Causes and outcomes of the acute chest syndrome in sickle cell disease. National Acute Chest Syndrome Study Group. N. Eng. J. Med..

[10] Styles LA, Aarsman AJ, Vichinsky EP, Kuypers FA (2000). Secretory phospholipase A (2) predicts impending acute chest syndrome in sickle cell disease. Blood..

[11] Ballas SK (2006). Secretory phospholipase A2 levels in patients with sickle cell disease and acute chest syndrome. Hemoglobin..

[12] Styles L (2012). Sickle Cell Disease Clinical Research Network (SCDCRN). Refining the value of secretory phospholipase A2 as a predictor of acute chest syndrome in sickle cell disease: results of a feasibility study (PROACTIVE). Br. J. Haematol..

[13] Nansseu JR (2015). The Acute Chest Syndrome in Cameroonian children living with sickle cell disease. BMC Pediatrics..

[14] Baum KF, Dunn DT, Maude GH, Serjeant GR (1987). The painful crisis of homozygous sickle cell disease. A study of the risk factors. Arch. Intern. Med..

[15] Platt OS (1994). Mortality in sickle cell disease, Life expectancy and risk factors for early death. N. Engl. J. Med..

[16] Gladwin MT (2003). Divergent nitric oxide bioavailability in men and women with sickle cell disease. Circulation..

[17] Ikuta T, Ausenda S, Cappellini MD (2001). Mechanism for fetal globin gene expression: role of the soluble guanylate cyclase-cGMP-dependent protein kinase pathway. Proc. Natl Acad. Sci. USA.

[18] Knight-Madden JM, Barton-Gooden A, Weaver SR, Reid M, Greenough A (2013). Mortality, asthma, smoking and acute chest syndrome in young adults with sickle cell disease. Lung..

[19] DeBaun MR (2014). Factors predicting future ACS episodes in children with sickle cell anemia. Am. J. Hematol..

[20] Castro O (1994). The acute chest syndrome in sickle cell disease: incidence and risk factors. The Cooperative Study of Sickle Cell Disease. Blood..

[21] Lazarus SG (2016). Are we missing the mark? Fever, respiratory symptoms, chest radiographs, and acute chest syndrome in sickle cell disease. Am. J. Hematol..

[22] Maitre Bernard, Habibi Anoosha, Roudot-Thoraval Françoise, Bachir Dora, Belghiti Dominique Desvaux, Galacteros Frederic, Godeau Bertrand (2000). Acute Chest Syndrome in Adults With Sickle Cell Disease. Chest.

[23] Allareddy V (2014). Outcomes of Acute Chest Syndrome in Adult Patients with Sickle Cell Disease: Predictors of Mortality. PLOS ONE..

